# Disparities in who is asked about their perinatal mental health: an analysis of cross-sectional data from consecutive national maternity surveys

**DOI:** 10.1186/s12884-023-05518-4

**Published:** 2023-04-27

**Authors:** Sian Harrison, Victoria Pilkington, Yangmei Li, Maria A. Quigley, Fiona Alderdice

**Affiliations:** 1 NIHR Policy Research Unit in Maternal and Neonatal Health and Care, National Perinatal Epidemiology Unit, Nuffield Department of Population Health, University of Oxford, Oxford, UK; 2 Oxford University Clinical Academic Graduate School, Oxford, UK

**Keywords:** Mental health, Pregnancy, Postnatal, Perinatal, Maternal Wellbeing, Disparities, Inequalities

## Abstract

**Background:**

The perinatal period is a vulnerable time, with one in five women experiencing mental health problems. Antenatal and postnatal appointments are key contact points for identifying women in need of support. Since 2014, the UK National Institute for Health and Care Excellence (NICE) has recommended that all women be asked about their mental health at their antenatal booking appointment and early in the postnatal period. The aim of this study was to assess the proportions of women who reported being asked about their mental health during the perinatal period across consecutive national maternity surveys (NMS) in England and to evaluate sociodemographic disparities in who was asked.

**Methods:**

Secondary analysis was performed on cross-sectional data from the NMS in 2014–2020. In each survey, women reported whether they had been asked about their mental health antenatally (during their booking appointment) and postnatally (up to six months after giving birth). The proportions of women in each survey who reported being asked about their mental health were calculated and compared according to key sociodemographic characteristics and across survey years. Logistic regression was conducted to identify disparities in who was asked.

**Results:**

The proportion of women who reported being asked about their mental health antenatally increased from 80.3% (95%CI:79.0–81.5) in 2014 to 83.4% (95%CI:82.1–84.7) in 2020, yet the proportion of women who reported being asked postnatally fell from 88.2% (95%CI:87.1–89.3) in 2014 to 73.7% (95%CI:72.2–75.2) in 2020. Ethnic minority women (aOR range:0.20 ~ 0.67) were less likely to report being asked about their mental health antenatally and postnatally across all surveys compared to White women. Women living in less socioeconomically advantaged areas (aOR range:0.65 ~ 0.75) and women living without or separately from a partner (aOR range:0.61 ~ 0.73) were also less likely to report being asked about their mental health, although there was less consistency in these disparities across the antenatal and postnatal periods and across surveys.

**Conclusions:**

Despite NICE recommendations, many women are still not asked about their mental health during the perinatal period, particularly after giving birth. Women from ethnic minority backgrounds are less likely to be asked and these disparities have persisted over time.

**Supplementary Information:**

The online version contains supplementary material available at 10.1186/s12884-023-05518-4.

## Background

Pregnancy and the postnatal period can be a time of stress and uncertainty for many women and their families. As many as one in five women develop a mental health problem during pregnancy or the first year after childbirth [1]. Mental health problems during pregnancy and the postnatal period can include a wide range of disorders, from anxiety [2] and depression [3], which are commonly comorbid, to more severe problems including posttraumatic stress disorder [4] mania and psychosis [5]. In addition to the potential onset of new problems during the perinatal period, pre-existing mental health problems may resurface, particularly if medication adjustments are required during pregnancy and breastfeeding. Women are therefore at risk of exacerbation of any pre-existing issues or relapse of previously well-controlled problems [6].

Perinatal mental health problems can lead to an increased risk of adverse outcomes including preterm birth, low birth weight, attachment difficulties, and longer term developmental difficulties for the child [7–10]. At the extreme, mental health problems contribute to maternal deaths, with suicide being one of the leading causes of maternal mortality in England [11, 12]. It is therefore essential that mental health is discussed with all women during their pregnancy and soon after childbirth.

In 2014, the National Institute for Health and Care Excellence (NICE) in the UK introduced a new guideline on Antenatal and Postnatal Mental Health, which recommends that a pregnant woman should be asked about her mental health and wellbeing at her first contact with primary care or at her antenatal booking appointment, and during the early postnatal period [13]. The guideline suggests standardised questions to use to identify possible depression or anxiety. Antenatal booking and the postnatal check appointments are key points of contact with healthcare services and they provide an opportunity to identify women with mental health problems, to offer support and, if appropriate, to refer to specialist mental health services. However, it is not known how widely the NICE guideline is being adhered to or to what extent disparities exist in the likelihood of women being asked about their mental health at these key contacts. Routinely asking women about their mental health during the perinatal period is the crucial first step in identifying those women who may need mental health support and treatment [14].

An analysis of data from the 2014 national maternity survey (NMS) in England found that almost one in five women were not asked about their mental health antenatally and one in ten were not asked postnatally [15]. Consequently, despite repeated contacts during the perinatal period, some women who were experiencing mental health problems would not have been identified. Furthermore, women from ethnic minority backgrounds, women living in less socioeconomically advantaged areas, and women who had completed fewer years of education were less likely to be asked [15]. These disparities are particularly concerning because women from ethnic minority and disadvantaged groups are at increased risk of adverse outcomes as a result of poor perinatal mental health [16–18].

The aim of this study was to estimate the proportions of women who reported being asked about their mental health during the perinatal period, as recommended in the NICE guideline, across three consecutive NMS in England (from 2014 to 2020) and to evaluate sociodemographic disparities in who was asked.

### Objectives

- To estimate the proportions of women who reported being asked about their mental health antenatally (during the booking appointment) and postnatally (up to six months after giving birth) across the 2014, 2018 and 2020 NMS
- To evaluate sociodemographic disparities in who reported being asked about their mental health antenatally and postnatally.

## Methods

### Study design and population

This study is a secondary analysis of cross-sectional data from three consecutive NMS carried out in England in 2014, 2018 and 2020. A detailed description of these surveys is included in the published reports for each survey year [19–21]. To summarise, women who gave birth during the selected time period of each survey year were identified via the Office for National Statistics (ONS) using birth registration records and were randomly chosen to be contacted for participation. Selected women (*N* = 10,000 in 2014, *N* = 16,000 in 2018 and *N* = 16,050 in 2020) were then contacted during the postnatal period and were sent the questionnaire, along with an invitation letter, an information leaflet and multi-language materials to enhance accessibility. For the 2014 survey, women were contacted three months after the birth of their baby, whereas for the 2018 and 2020 surveys, contact was six months after birth. Women aged less than 16 years old or whose baby had died were excluded from the survey mailings. Questionnaire participation was via a paper form to be filled in and returned by freepost, or alternatively the questionnaire could be completed online. Up to three reminders were sent out to women who had been selected to take part.

### Measures

The questionnaires included questions about events, care and experience during pregnancy, birth and the postnatal period, as well as sociodemographic characteristics of the respondents, including ethnicity, level of education, relationship status (living with a partner or not) and parity. Additional sociodemographic data were provided by ONS, including women’s age, country of birth, and an Index of Multiple Deprivation (IMD) measure based on area of residence. The outcome of interest for this study was whether women were asked about their mental health antenatally and postnatally; these time points were assessed separately. Three questions were identified as being relevant and consistent across survey years (see Additional file 1 for the specific questions and response options in each survey year).

1. Around the time of your pregnancy booking, were you asked about your mental or emotional health?
2. Around the time of your pregnancy booking, were you asked about your past mental health or family history of mental health problems?
3. Since your baby was born, have you been asked about your mental or emotional health?

It is important to note that the outcome data were self-reported and were not verified and so rely on women’s recollection, understanding and reporting of discussions held at their antenatal and postnatal appointments. It is also important to note that women who took part in the 2020 NMS would have had their postnatal appointments during the Covid-19 pandemic and may have had some of their antenatal appointments (including their booking appointment if it was particularly late in their pregnancy) during the pandemic.

### Analysis

ONS provided sociodemographic data for all women who were selected for each of the survey years, allowing comparison of respondents and non-respondents. In all survey years, response rates were higher for women who were older, who registered the birth of their baby in married names, who were born in the UK, and who lived in more socioeconomically advantaged areas [19–21]. In the 2018 and 2020 surveys, response rates were also higher for women who had not given birth before. These sociodemographic data were used to calculate survey weights for each survey year individually [19–21] and the survey weights were applied to the data in the current analysis.

Sociodemographic variables were coded into the following pre-set categories: age group (16–19 years, 20–24 years, 25–29 years, 30–34 years, 35–39 years, 40 + years); ethnic group (White, Mixed, Asian, Black, Other); IMD quintile (1 = least socioeconomically advantaged, 5 = most socioeconomically advantaged); relationship status (living with a partner or not); education level (left full-time education at ≤ 16 years, 17–18 years, ≥ 19 years); and parity (primiparous, multiparous). Each question outcome variable was coded into binary response options (yes or no) to allow binary logistic regression analysis to be performed. In order to make results comparable across the surveys, some response options were recoded into affirmative (yes) and non-affirmative (no and don’t know) (see Additional file 1).

A descriptive analysis was first carried out for each of the three question outcome variables using overall percentage proportions and 95% confidence intervals (CI) to estimate the proportion of women who were asked about their mental health antenatally and postnatally and to establish how well the NICE guideline was being followed. The proportions were compared across survey years with Chi-Square test for trend used to determine if there were any statistically significant trends over time. Further descriptive analysis by key sociodemographic variables was then performed to explore potential disparities in who was asked about their mental health. Proportions were calculated for each category of sociodemographic variables, and univariable and multivariable logistic regression models were used to explore the association between women’s characteristics and whether they were asked about their mental health. Odds ratios (OR) and adjusted ORs (aOR) are reported with 95% CIs. Each survey year was analysed separately and all analyses were carried out in STATA 17.6 [22].

## Results

### Characteristics of respondents

Completed survey returns were received from 4,571 (46.7%) women in 2014, 4,509 (29.0%) women in 2018 and 4,611 (28.9%) women in 2020. Overall response rates declined across survey years for all sociodemographic groups [23]. The distributions of the sociodemographic characteristics of respondents were similar across survey years (Table 1). Compared to the non-respondents, the respondents in each survey were more likely to be older, to self-identify as being of White ethnicity, to be living in more socioeconomically advantaged areas, to have left full-time education at 19 years of age or older, and to be living with a partner at the time they participated in the survey. Similar proportions of primiparous and multiparous women responded in each survey. There were some changes in the characteristics of respondents over time, for example small decreases in the proportions of women from younger age groups, women from ethnic minority backgrounds, and women living in less socioeconomically advantaged areas.

**Table 1:** Characteristics of respondents by year

	All births in England in 2020	2014 survey *N* = 4571		2018 survey *N* = 4509		2020 survey *N* = 4611	
	**%**	**n**	**%**	**n**	**%**	**n**	**%**
**Age**	**^**						
16–19 years	2.6	101	2.2	59	1.3	44	1.0
20–24 years	13.0	538	11.8	359	8.0	355	7.7
25–29 years	26.8	1228	26.9	1055	23.4	1117	24.2
30–34 years	33.5	1587	34.7	1713	38.0	1785	38.7
35–39 years	19.3	874	19.1	1054	23.4	1089	23.6
40 + years	4.8	241	5.3	269	6.0	221	4.8
*Missing*	*0.0*	*2*	*0.0*	*0*	*0.0*	*0*	*0.0*
**Ethnicity** ^a^							
White	72.4	3710	83.9	3779	86.7	3911	86.0
Mixed	7.0	87	2.0	101	2.3	104	2.3
Asian	13.0	442	10.0	308	7.1	380	8.4
Black	5.2	159	3.6	102	2.3	126	2.8
Other	2.4	23	0.5	67	1.5	27	0.6
*Missing*	*2.4*	*150*	*3.3*	*152*	*3.4*	*63*	*1.4*
**IMD**							
1 (least advantaged)	25.6	893	19.6	706	15.7	698	15.1
2	22.4	977	21.4	869	19.3	876	19.0
3	19.5	934	20.5	945	21.0	957	20.8
4	17.3	865	18.9	1006	22.3	1070	23.2
5 (most advantaged)	15.3	899	19.7	983	21.8	1010	21.9
*Missing*	*0.0*	*3*	*0.1*	*0*	*0.0*	*0*	*0.0*
**Education**							
< 17 years	NA	756	16.9	493	11.1	514	11.3
17–18 years	NA	1209	27.0	1045	23.4	1226	26.9
19 + years	NA	2509	56.0	2922	65.5	2823	61.9
*Missing*		*97*	*2.1*	*49*	*1.1*	*48*	*1.0*
**Relationship Status**							
Lives with partner	84.6	3980	87.1	4045	89.7	4144	89.9
Does not live with partner	15.4	591	12.9	464	10.3	467	10.1
*Missing*	*0.0*	*0*	*0.0*	*0*	*0.0*	*0*	*0.0*
**Parity**	^						
Primiparous	44.2	2206	49.9	2331	52.8	2174	48.2
Multiparious	55.8	2217	50.1	2088	47.3	2333	51.8
*Missing*	*0.0*	*148*	*3.2*	*90*	*2.0*	*104*	*2.3*

Proportions are presented as the % of non-missing

^Data for England and Wales combined

^+^Based on registration status (yes = registered in married names or joint names, same address) for population-level data

^a^Ethnicity relates to baby for population-level data and to mother for survey data

### Proportion of women who were asked about their mental health during the perinatal period

**Fig. 1:**
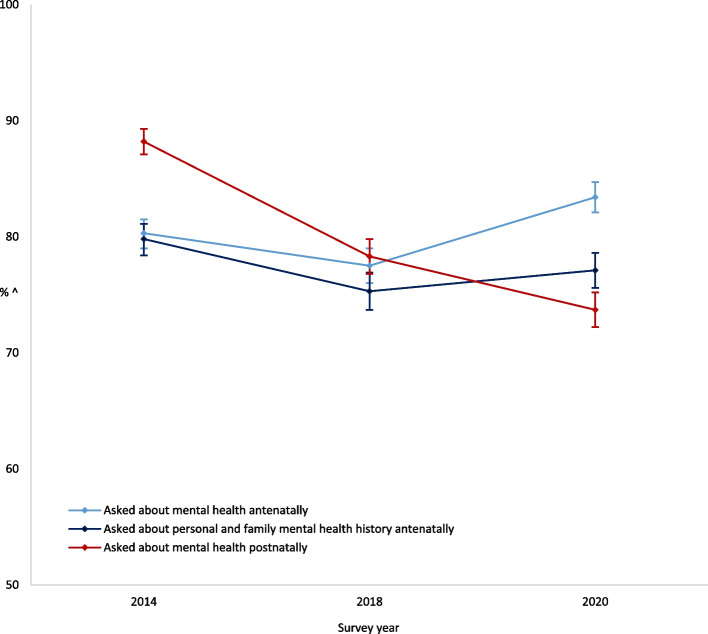
Proportions of women who were asked about their mental health antenatally and postnatally across survey years. ^The Y axis starts at 50%

Overall, the proportion of women who reported being asked about their current mental health at their antenatal booking appointment decreased from 80.3% (95%CI: 79.0–81.5) in 2014 to 77.5% (95%CI: 76.0–79.0) in 2018 and then increased again to 83.4% (95%CI: 82.1–84.7) in 2020. The proportion of women who reported being asked about their mental health history at their antenatal booking appointment decreased from 79.8% (95%CI: 78.4–81.1) in 2014 to 75.3% (95%CI: 73.7–76.9) in 2018 and then increased again to 77.1% (95%CI: 75.6–78.6) in 2020. The proportion of women who reported being asked about their mental health during the postnatal period decreased from 88.2% (95%CI: 87.1–89.3) in 2014 to 78.3% in 2018 (95%CI: 76.8–79.8) and then decreased further to 73.7% (95%CI: 72.2–75.2) in 2020 (Tables 2, 3 and 4, Fig. 1).

**Table 2:** Association between women’s sociodemographic characteristics and being asked about mental health during the antenatal booking appointment across surveys

	**2014 survey** ***N*** ** = 4571**						**2018 survey** ***N*** ** = 4509**						**2020 survey** ***N*** ** = 4611**					
	**n**	**%**	**OR**	**95%CI**	**aOR** ^a^	**95% CI**	**n**	**%**	**OR**	**95%CI**	**aOR** ^a^	**95% CI**	**n**	**%**	**OR**	**95%CI**	**aOR** ^a^	**95% CI**
***Total n % (95%CI)***	***3562***	***80.3***	***(79.0, 81.5)***				***3600***	***77.5***	***(76.0, 79.0)***				***3891***	***83.4***	***(82.1, 84.7)***			
**Age of Mother**																		
16–19	78	79.1	0.90	0.52, 1.56	0.87	0.47, 1.59	46	76.1	0.86	0.43, 1.74	1.26	0.54, 2.96	39	89.5	1.80	0.67, 4.80	1.64	0.60, 4.49
20–24	403	79.4	0.92	0.70, 1.19	0.91	0.68, 1.21	281	75.8	0.85	0.62, 1.17	1.01	0.70, 1.44	307	85.5	1.24	0.84, 1.82	1.23	0.81, 1.86
25–29	982	81.9	1.08	0.88, 1.33	1.09	0.87, 1.35	854	77.0	0.91	0.72, 1.14	0.93	0.73, 1.19	972	85.9	1.28	1.00, 1.65	1.37	1.06, 1.78
30–34	1248	80.8			1		1380	78.7	1		1		1498	82.6	1		1	
35–39	679	79.3	0.91	0.73, 1.13	0.88	0.70, 1.10	830	77.3	0.92	0.74, 1.15	0.95	0.75, 1.19	902	80.9	0.89	0.71, 1.13	0.89	0.70, 1.14
40 +	173	74.8	**0.70**	**0.50, 0.99**	0.76	0.53, 1.08	209	78.6	1.00	0.70, 1.42	0.97	0.67, 1.39	173	76.3	0.68	0.45, 1.01	0.75	0.49, 1.15
**Ethnicity**																		
White	2951	81.7	1		1		3106	81.1	1		1		3373	86.3	1		1	
Mixed	67	79.8	0.89	0.48, 1.65	0.89	0.47, 1.65	82	77.4	0.80	0.44, 1.45	0.87	0.48, 1.57	85	82.2	0.73	0.41, 1.32	0.85	0.45, 1.61
Asian	311	73.2	**0.61**	**0.48, 0.79**	**0.65**	**0.50, 0.85**	204	62.3	**0.39**	**0.29, 0.52**	**0.39**	**0.29, 0.53**	276	68.7	**0.35**	**0.27, 0.46**	**0.42**	**0.32, 0.56**
Black	121	79.6	0.87	0.56, 1.36	0.99	0.60, 1.60	70	65.8	**0.45**	**0.27, 0.74**	0.60	0.35, 1.04	92	75.4	**0.49**	**0.30, 0.78**	0.64	0.38, 1.07
Other	17	78.0	0.80	0.29, 2.21	0.82	0.29, 2.32	48	72.5	0.61	0.33, 1.16	0.63	0.32, 1.20	17	63.3	**0.27**	**0.12, 0.64**	**0.33**	**0.13, 0.86**
**IMD**																		
1 (least advantaged)	664	78.2	0.93	0.73, 1.18	1.05	0.81, 1.37	528	71.6	**0.59**	**0.46, 0.76**	**0.75**	**0.57, 0.98**	575	81.9	0.76	0.57, 1.02	0.82	0.60, 1.12
2	772	81.2	1.12	0.88, 1.42	1.22	0.95. 1.57	694	77.1	0.78	0.61, 1.01	0.99	0.76, 1.30	726	80.7	**0.70**	**0.53, 0.93**	0.78	0.58, 1.05
3	742	81.6	1.15	0.90, 1.46	1.19	0.93, 1.52	764	80.1	0.94	0.73, 1.20	1.03	0.79, 1.33	812	84.3	0.91	0.69, 1.19	0.92	0.69, 1.22
4	693	82.0	1.18	0.92, 1.50	1.24	0.96, 1.60	824	81.5	1.03	0.81, 1.31	1.12	0.86, 1.44	915	86.1	1.04	0.80, 1.35	1.00	0.76, 1.32
5 (most advantaged)	691	79.4	1		1		790	81.1	1		1		863	85.6	1		1	
**Education**																		
< 17	569	79.2	0.88	0.71, 1.10	0.91	0.71, 1.17	377	73.6	0.77	0.59, 1.01	0.81	0.60, 1.09	435	85.2	1.32	0.97, 1.79	1.29	0.92, 1.83
17–18	948	80.1	0.93	0.77, 1.13	0.91	0.74, 1.13	857	79.3	1.06	0.85, 1.32	1.02	0.80, 1.29	1066	87.1	**1.54**	**1.23, 1.93**	**1.37**	**1.06, 1.76**
19 +	1989	81.2	1		1		2342	78.4	1		1		2356	81.4	1		1	
**Lives with partner**																		
No	432	78.3	0.86	0.68, 1.09	0.90	0.69, 1.18	330	68.6	**0.56**	**0.44, 0.72**	**0.67**	**0.50, 0.89**	377	79.9	0.75	0.56, 1.00	**0.70**	**0.50, 0.97**
Yes	3133	80.7	1		1		3270	79.5	1		1		3514	84.2	1		1	
**Parity**																		
Primiparous	1754	81.6	**1.19**	**1.01, 1.41**	1.12	0.94, 1.34	1910	80.1	**1.31**	**1.10, 1.56**	1.21	1.00, 1.46	1989	83.0	1.11	0.92, 1.35	0.97	0.80, 1.19
Multiparous	1707	78.8	1		1		1625	75.5	1		1		1820	85.5	1		1	

2014 survey data weighted by age, country of birth, registration status and IMD quintile

2018 survey data weighted by age, country of birth, registration status, IMD quintile, region of residence and parity

2020 survey data weighted by age, country of birth, registration status, IMD quintile, region of residence and parity

^a^Variables mutually adjusted in the multivariable analysis

**Table 3:** Association between women’s sociodemographic characteristics and being asked about personal and family history of mental health problems during the antenatal booking appointment across surveys

	**2014 survey** ***N*** ** = 4571**						**2018 survey** ***N*** ** = 4509**						**2020 survey** ***N*** ** = 4611**					
	**n**	**%**	**OR**	**95%CI**	**aOR** ^a^	**95% CI**	**n**	**%**	**OR**	**95%CI**	**aOR** ^a^	**95% CI**	**n**	**%**	**OR**	**95%CI**	**aOR** ^a^	**95% CI**
***Total n % (95%CI)***	***3082***	***79.8***	***(78.4, 81.1)***				***3509***	***75.3***	***(73.7, 76.9)***				***3637***	***77.1***	***(75.6, 78.6)***			
**Age of Mother**																		
16–19	70	79.7	1.01	0.56, 1.81	0.99	0.53, 1.85	50	77.8	1.13	0.52, 2.47	1.21	0.49, 2.99	36	79.9	1.10	0.47, 2.56	1.23	0.50, 3.04
20–24	359	81.3	1.11	0.83, 1.50	1.17	0.85, 1.60	280	76.3	1.03	0.75, 1.42	1.15	0.79, 1.65	294	82.1	1.27	0.89, 1.80	1.22	0.83, 1.79
25–29	852	82.2	1.18	0.95, 1.48	1.21	0.95, 1.52	845	76.1	1.02	0.81, 1.28	1.08	0.85, 1.38	896	77.5	0.95	0.76, 1.18	0.95	0.76, 1.19
30–34	1076	79.6	1		1		1342	75.7	1		1		1430	78.3	1		1	
35–39	585	77.4	0.88	0.70, 1.10	0.80	0.63, 1.01	796	73.4	0.89	0.72, 1.10	0.94	0.76, 1.17	819	73.1	**0.75**	**0.61, 0.93**	**0.75**	**0.60, 0.94**
40 +	141	69.5	**0.58**	**0.42, 0.82**	**0.58**	**0.41, 0.83**	196	72.6	0.85	0.62, 1.18	0.84	0.60, 1.19	162	68.2	**0.59**	**0.41, 0.85**	0.70	0.48, 1.02
**Ethnicity**																		
White	2559	81.4	1		1		3060	80.4	1		1		3207	81.8	1		1	
Mixed	54	73.9	0.64	0.34, 1.24	0.63	0.33, 1.20	80	80.5	1.00	0.55, 1.84	1.14	0.62, 2.09	69	62.1	**0.36**	**0.23, 0.58**	**0.40**	**0.24, 0.64**
Asian	272	72.7	**0.61**	**0.47, 0.79**	**0.61**	**0.46, 0.81**	185	54.1	**0.29**	**0.22, 0.38**	**0.32**	**0.24, 0.43**	234	57.5	**0.30**	**0.23, 0.39**	**0.35**	**0.27, 0.45**
Black	101	77.4	0.78	0.51, 1.20	0.91	0.55, 1.48	62	58.6	**0.34**	**0.22, 0.55**	**0.49**	**0.30, 0.82**	75	60.0	**0.33**	**0.21, 0.52**	**0.49**	**0.31, 0.77**
Other	14	75.0	0.68	0.24, 1.93	0.70	0.23, 2.08	40	56.3	**0.31**	**0.17, 0.56**	**0.33**	**0.18, 0.59**	14	54.9	**0.27**	**0.12, 0.63**	**0.31**	**0.12, 0.75**
**IMD**																		
1 (least advantaged)	561	78.5	1.00	0.77, 1.29	1.19	0.90, 1.58	521	68.9	**0.55**	**0.42, 0.70**	**0.75**	**0.57, 0.98**	520	73.2	**0.72**	**0.56, 0.92**	0.82	0.62, 1.08
2	659	80.5	1.13	0.88, 1.45	1.30	0.99, 1.70	683	75.9	**0.78**	**0.61, 0.99**	0.98	0.75, 1.28	685	75.0	0.79	0.61, 1.01	0.91	0.70, 1.19
3	638	80.4	1.12	0.87, 1.44	1.15	0.89, 1.49	735	76.7	0.81	0.64, 1.03	0.90	0.70, 1.15	768	80.4	1.07	0.84, 1.37	1.13	0.88, 1.45
4	611	81.4	1.20	0.93, 1.55	1.20	0.92, 1.56	787	79.0	0.93	0.74, 1.17	0.99	0.77, 1.27	858	79.9	1.04	0.82, 1.31	0.99	0.77, 1.26
5 (most advantaged)	613	78.5	1		1		783	80.2	1		1		806	79.2	1		1	
**Education**																		
< 17	468	77.4	0.84	0.66, 1.05	**0.77**	**0.59, 0.99**	379	73.1	0.92	0.71, 1.21	1.00	0.74, 1.34	408	79.4	1.28	0.98, 1.68	1.17	0.87, 1.58
17–18	833	81.0	1.04	0.85, 1.27	0.93	0.75, 1.16	861	80.3	**1.38**	**1.11, 1.73**	**1.38**	**1.08, 1.76**	1010	81.5	**1.47**	**1.19, 1.80**	1.24	0.99, 1.54
19 +	1735	80.4	1		1		2247	74.7	1		1		2191	75.1	1		1	
**Lives with partner**																		
No	367	76.5	0.79	0.61, 1.01	**0.73**	**0.55, 0.97**	317	66.2	**0.57**	**0.45, 0.73**	**0.61**	**0.46, 0.80**	353	73.6	0.79	0.61, 1.04	0.87	0.64, 1.18
Yes	2718	80.5	1		1		3192	77.4	1		1		3284	77.9	1		1	
**Parity**																		
Primiparous	1524	81.3	1.17	0.98, 1.39	1.03	0.86, 1.24	1877	79.1	**1.45**	**1.22, 1.72**	**1.32**	**1.09, 1.59**	1872	78.7	1.17	0.98, 1.39	1.08	0.90, 1.29
Multiparous	1480	78.8	1		1		1570	72.3	1		1		1684	76.0	1		1	

2014 survey data weighted by age, country of birth, registration status and IMD quintile

2018 survey data weighted by age, country of birth, registration status, IMD quintile, region of residence and parity

2020 survey data weighted by age, country of birth, registration status, IMD quintile, region of residence and parity

^a^Variables mutually adjusted in the multivariable analysis

**Table 4:** Association between women’s sociodemographic characteristics and being asked about mental health during the postnatal check appointment across surveys

	**2014 survey** ***N*** ** = 4571**						**2018 survey** ***N*** ** = 4509**						**2020 survey** ***N*** ** = 4611**					
	**n**	**%**	**OR**	**95%CI**	**aOR** ^a^	**95% CI**	**n**	**%**	**OR**	**95%CI**	**aOR** ^a^	**95% CI**	**n**	**%**	**OR**	**95%CI**	**aOR** ^a^	**95% CI**
***Total n % (95%CI)***	***4035***	***88.2***					***3652***	***78.3***	***(76.8, 79.8)***				***3438***	***73.7***	***(72.2, 75.2)***			
**Age of Mother**																		
16–19	79	79.1	**0.42**	**0.24, 0.73**	**0.35**	**0.19, 0.65**	46	80.0	0.92	0.46, 1.82	0.98	0.44, 2.14	37	87.4	2.16	0.92, 5.08	2.14	0.89, 5.15
20–24	457	86.1	**0.68**	**0.49, 0.95**	0.73	0.50, 1.06	251	69.8	**0.53**	**0.40, 0.71**	**0.60**	**0.43, 0.83**	245	69.5	**0.71**	**0.54, 0.94**	0.77	0.57, 1.04
25–29	1082	88.3	0.84	0.64, 1.10	0.82	0.61, 1.09	837	76.0	**0.73**	**0.58, 0.92**	**0.78**	**0.61, 0.99**	790	68.7	**0.69**	**0.57, 0.83**	**0.71**	**0.58, 0.87**
30–34	1426	90.0	1		1		1426	81.3	1		1		1347	76.2	1		1	
35–39	780	89.4	0.94	0.69, 1.27	0.90	0.66, 1.24	862	81.0	0.98	0.78, 1.23	1.01	0.79, 1.29	839	75.2	0.95	0.78, 1.16	0.94	0.77, 1.16
40 +	212	87.4	0.77	0.48, 1.22	0.72	0.45, 1.15	230	84.4	1.24	0.83, 1.86	1.34	0.87, 2.07	180	80.9	1.33	0.89, 1.98	1.35	0.88, 2.07
**Ethnicity**																		
White	3394	91.2	1		1		3121	81.2	1		1		2945	74.8	1		1	
Mixed	74	83.9	**0.50**	**0.26, 0.97**	**0.50**	**0.26, 0.98**	80	70.8	**0.56**	**0.32, 0.99**	**0.56**	**0.33, 0.96**	73	65.9	0.65	0.40, 1.06	0.63	0.38, 1.03
Asian	348	75.3	**0.29**	**0.22, 0.39**	**0.28**	**0.21, 0.38**	226	69.2	**0.52**	**0.38, 0.71**	**0.55**	**0.39, 0.76**	256	66.6	**0.67**	**0.52, 0.87**	**0.67**	**0.51, 0.87**
Black	124	80.7	**0.40**	**0.26, 0.62**	**0.43**	**0.26, 0.71**	72	67.4	**0.48**	**0.29, 0.81**	**0.55**	**0.32, 0.95**	99	75.7	1.05	0.63, 1.75	0.98	0.59, 1.64
Other	16	69.6	**0.22**	**0.09, 0.55**	**0.20**	**0.08, 0.50**	53	74.0	0.66	0.33, 1.32	0.52	0.26, 1.04	17	64.1	0.60	0.25, 1.47	0.63	0.26, 1.54
**IMD**																		
1 (least advantaged)	726	82.3	**0.39**	**0.29, 0.54**	**0.69**	**0.48, 0.98**	524	71.4	**0.47**	**0.36, 0.62**	**0.65**	**0.49, 0.88**	493	71.0	0.81	0.63, 1.02	0.92	0.72, 1.18
2	858	88.0	**0.62**	**0.44, 0.86**	0.99	0.69, 1.41	694	77.5	**0.65**	**0.50, 0.85**	0.88	0.66, 1.17	653	74.0	0.94	0.75, 1.18	1.05	0.83, 1.33
3	843	91.2	0.88	0.62, 1.25	1.07	0.74, 1.54	773	82.0	0.87	0.67, 1.13	1.00	0.76, 1.31	719	74.1	0.94	0.75, 1.17	0.99	0.79, 1.25
4	785	91.4	0.90	0.63, 1.29	1.01	0.69, 1.47	829	81.3	0.83	0.64, 1.07	0.87	0.66, 1.13	816	75.6	1.02	0.82, 1.27	1.01	0.81, 1.26
5 (most advantaged)	823	92.2	1		1		832	84.1	1		1		757	75.2	1		1	
**Education**																		
< 17	661	86.4	**0.69**	**0.52, 0.91**	**0.66**	**0.49, 0.90**	375	74.4	**0.70**	**0.53, 0.91**	0.80	0.59, 1.09	353	69.4	**0.72**	**0.57, 0.92**	**0.73**	**0.57, 0.94**
17–18	1056	86.4	**0.68**	**0.54, 0.87**	**0.62**	**0.47, 0.81**	820	76.3	**0.77**	**0.63, 0.96**	0.84	0.66, 1.06	889	71.4	**0.80**	**0.67, 0.95**	**0.82**	**0.68, 0.99**
19 +	2282	90.3	1		1		2426	80.6	1		1		2165	75.8	1		1	
**Lives with partner**																		
No	456	83.7	**0.62**	**0.47, 0.81**	0.77	0.56, 1.05	324	69.9	**0.57**	**0.45, 0.74**	0.80	0.59, 1.07	330	72.2	0.91	0.71, 1.16	0.95	0.73, 1.24
Yes	3582	83.7	1		1		3328	80.2	1		1		3108	74.0	1		1	
**Parity**																		
Primiparous	1999	89.5	**1.27**	**1.03, 1.58**	1.24	0.98, 1.56	1926	81.1	**1.34**	**1.12, 1.60**	**1.27**	**1.04, 1.54**	1754	74.2	1.05	0.90, 1.23	1.04	0.86, 1.23
Multiparous	1952	87.0	1		1		1663	76.2	1		1		1604	73.2	1		1	

2014 survey data weighted by age, country of birth, registration status and IMD quintile

2018 survey data weighted by age, country of birth, registration status, IMD quintile, region of residence and parity

2020 survey data weighted by age, country of birth, registration status, IMD quintile, region of residence and parity

^a^Variables mutually adjusted in the multivariable analysis

### Disparities in who was asked about their mental health antenatally

Table 2 shows the proportions of women who were asked about their current mental health during the antenatal booking appointment according to different sociodemographic characteristics and across survey years. Crude and adjusted ORs for the association between sociodemographic characteristics and antenatal mental health assessment are also shown. Asian women were less likely to be asked about their current mental health at their antenatal booking appointment when compared to White women across all survey years (aORs ranged from 0.39 to 0.65). In the 2020 survey, women from Other ethnic minority backgrounds were also less likely to be asked than White women (63.3% vs. 86.3%, aOR: 0.33, 95%CI: 0.13–0.86). In the 2018 and 2020 surveys, women living in the least socioeconomically advantaged areas (IMD = 1 or 2) were less likely to be asked about their current mental health antenatally compared to women living in the most socioeconomically advantaged areas (IMD = 5) but, after adjusting for other variables, the association was only statistically significant in the 2018 survey (71.6% vs. 81.1%, aOR: 0.75, 95%CI: 0.57–0.98). Women who were living without or separately from a partner were less likely to be asked about their current mental health antenatally in the 2018 survey (68.6% vs. 79.5%, aOR: 0.67, 95%CI: 0.50–0.89) and in the 2020 survey (79.9% vs. 84.2%, aOR: 0.70, 95%CI: 0.50–0.97). In the 2020 survey, women who left education before the age of 19 years were more likely to be asked about their current mental health antenatally, yet the association was only statistically significant for those women leaving education aged 17 or 18 years (87.1% vs. 81.4%, aOR: 1.37, 95%CI: 1.06–1.76), and not for the women who left education at an even younger age (85.2% vs. 81.4%, aOR: 1.29, 95%CI: 0.92–1.83). There was no evidence of association between women’s age or parity and being asked about their mental health antenatally in the 2020 survey, or in the earlier surveys after adjusting for other variables.

Table 3 shows the proportions of women who were asked about their mental health history during the antenatal booking appointment according to different sociodemographic characteristics and across survey years. Crude and adjusted ORs for the association between sociodemographic characteristics and antenatal mental health history assessment are also shown. Compared to women aged 30–34 years (the reference group), women aged 40 years and over were less likely to be asked in the 2014 survey (69.5% vs. 79.6%, aOR: 0.58, 95%CI: 0.41–0.83), and women aged 35–39 years were less likely to be asked in the 2020 survey (73.1% vs. 78.3%, aOR: 0.75, 95%CI: 0.60–0.94). Asian women were less likely to be asked about their mental health history compared to White women in the 2014 survey (72.% vs. 81.4%, aOR: 0.61, 95%CI: 0.46–0.81). In the 2018 survey, Black women (58.6%, aOR: 0.49, 95%CI: 0.30–0.82), Asian women (54.1%, aOR: 0.32, 95%CI: 0.24–0.43) and women from Other ethnic minority backgrounds (56.3%, aOR: 0.33, 95%CI: 0.18–0.59) were also less likely to be asked compared to White women (80.4%). In the 2020 survey, Black women (60.0%, aOR: 0.49, 95%CI: 0.31–0.77), Asian women (57.5%, aOR: 0.35, 95%CI: 0.27–0.45), women of Mixed ethnicity (62.1%, aOR: 0.40, 95%CI: 0.24–0.64), and women from Other ethnic minority backgrounds (54.9%, aOR: 0.31, 95%CI: 0.12–0.75) were all less likely to be asked about their mental health history antenatally compared to White women (81.8%). In the 2018 and 2020 surveys, women who were living in the least socioeconomically advantaged areas (IMD = 1) were less likely to be asked compared to women living in the most socioeconomically advantaged areas (IMD = 5) but, after adjusting for other variables, the association was only statistically significant in the 2018 survey (68.9% vs. 80.2%, aOR: 0.75, 95%CI: 0.57–0.98). Compared to women who left education at 19 years of age or older, women who left education at 16 years of age or younger were less likely to be asked in the 2014 survey (77.4% vs. 80.4%, aOR: 0.77, 95%CI: 0.59–0.99), and women who left education aged 17 or 18 years were more likely to be asked in the 2018 survey (80.3% vs. 74.7%, aOR: 1.38, 95%CI: 1.08–1.76). Women living without or separately from a partner were less likely to be asked about their mental health history antenatally in the 2014 survey (76.5% vs. 80.5%, aOR: 0.73, 95%CI: 0.55–0.97) and the 2018 survey (66.2% vs. 77.4%, aOR: 0.61, 95%CI: 0.46–0.80). In the 2018 survey, primiparous women were more likely to be asked compared to multiparous women (79.1% vs. 72.3%, aOR: 1.32, 95% 1.09–1.59).

### Disparities in who was asked about their mental health postnatally

Table 4 shows the proportions of women who were asked about their mental health during the postnatal period according to different sociodemographic characteristics and across survey years. Crude and adjusted ORs for the association between sociodemographic characteristics and being asked about postnatal mental health are also shown. Younger women (aged < 30 years) were less likely to be asked about their mental health postnatally when compared to women who were aged 30–34 years (reference group) across all surveys (aORs ranged from 0.35 to 0.78), although statistical significance varied depending on the younger age group categories. In the 2014 and 2018 surveys, Black, Asian, and Mixed ethnicity women were less likely to be asked about their mental health postnatally compared to White women (aORs ranged from 0.20 to 0.56), yet in the 2020 survey the difference was only statistically significant for Asian women compared to White women (66.6% vs. 74.8%, aOR: 0.67, 95%CI: 0.51–0.87). Women living in the least socioeconomically advantaged areas (IMD = 1) were less likely to be asked about their mental health postnatally compared to those living in the most socioeconomically advantaged areas (IMD = 5) in the 2014 survey (82.3% vs. 92.2%, aOR: 0.69, 95%CI: 0.48–0.98) and the 2018 survey (71.4% vs. 84.1%, aOR: 0.65, 95%CI: 0.49–0.88), yet there was no evidence of association between IMD and being asked about mental health postnatally in the 2020 survey. Women who left education before 19 years of age were less likely to be asked about their mental health postnatally compared to women who left education aged 19 years or older (aORs ranged from 0.62 to 0.84) but, after adjusting for other variables, the association was not statistically significant in the 2018 survey. In the 2018 survey, primiparous women were more likely to be asked about their mental health postnatally compared to multiparous women (81.1% vs. 76.2%, aOR: 1.27, 95%CI: 1.04–1.54). There was no evidence that living with a partner was associated with being asked about mental health postnatally after adjusting for other variables in any of the surveys.

## Discussion

### Summary of findings

This analysis provides insight into whether women who gave birth in England from 2014 to 2020 were asked about their mental health during the perinatal period, as recommended in the NICE guideline since 2014. Whilst the proportion of women who reported being asked about their mental health at their pregnancy booking appointment rose between the 2014 and 2020 surveys, one in six women in the 2020 survey were still not asked about their mental health at this key antenatal contact. Furthermore, the proportion of women who were asked about their mental health during the postnatal period fell between 2014 and 2020, with more than a quarter of women not being asked about their mental health following childbirth. The perinatal period is a vulnerable time for women; failure to comply with the NICE guideline and the large number of women who are not undergoing even a basic mental health screen during pregnancy and after giving birth is a significant concern.

The findings from the current study are consistent with those from a 2017 survey conducted by the UK Royal College of Obstetricians and Gynaecologists (RCOG), which found that over a quarter of the 2,300 postnatal women sampled reported never being asked about their mental health during pregnancy [24]. Conversely, the 2021 maternity survey carried out by the Care Quality Commission (CQC) found that over 90% of women were asked about their mental health during their antenatal and postnatal appointments, similar proportions to in the 2019 CQC maternity survey [25]. It is important to interpret the 2020 survey findings in the current study within the context of the pandemic. While the majority of women who took part would have had their pregnancy booking appointment in late 2019, prior to the pandemic, all women would have been due their postnatal checks during the first wave of Covid-19. We know that maternity services were curtailed during this time with reduced midwifery visits and face-to-face contact [19]. There were also fewer postnatal checks carried out in 2020. In the UK, postnatal maternal checks are carried out in the community by General Practitioners (GPs) and in February 2020 their contract was updated to include incentives for the completion of these checks, within which a review of mental health and general wellbeing is specified [26]. Despite this, fewer women who gave birth during the first wave of the pandemic in the UK were followed-up by their GP compared to women who gave birth pre-pandemic [19].

Despite the variation in findings between the different surveys, they all indicate that at least a significant minority of women are not asked about their mental health during the perinatal period. Several qualitative studies have explored midwives’ views of mental health screening [27–29]. The findings offer a number of explanations for why women may not be asked about their mental health. These include high workload [27] and time constraints within appointments, particularly the antenatal booking appointment where a lot of information gathering and procedures have to be carried out [28, 29]. In addition, some midwives considered the booking appointment too early in pregnancy at a time when they did not know the women well [28]. Midwives also reported occasionally avoiding the assessment as it felt intrusive or they felt questions could be perceived as quite blunt or unclear [28]. Uncertainty about how to support women who screened positive due to perceived limited availability of treatment options, and lack of mental health expertise and confidence, particularly amongst junior midwives, were also cited as challenges in contemporary practice which may impede asking women about their mental health [27–29].

The current study indicates that women from ethnic minority backgrounds were less likely to be asked about their mental health during the perinatal period compared to White women and this finding has persisted over time. Women from ethnic minority backgrounds were less likely to be asked both during their pregnancy and after childbirth, although the gap postnatally narrowed in the 2020 survey. This finding is consistent with the results of a secondary analysis of the Born in Bradford data which found that women from ethnic minority backgrounds were half as likely to have screening, and twice as likely to have a mental health problem missed as White British women [30]. One explanation for this disparity is that healthcare professionals may not have adequate conversations surrounding mental health with women from ethnic minority backgrounds. In a recent qualitative study, midwives reported discomfort asking women from ethnic minority backgrounds the screening questions recommended in the NICE guideline, because they felt that mental illness is not always recognised in the same capacity as physical illness within certain cultures [30]. They also reported concern that women from ethnic minority backgrounds may feel greater stigma when discussing mental health problems, due to cultural beliefs [30]. Another possible reason that healthcare professionals may resist discussions about mental health with women from ethnic minority backgrounds is potential language barriers, which could compound the challenges of time constraints and lack of confidence. Language barriers might also affect women’s understanding of questions regarding their mental health or their association of the questions with mental health. For example, women may use different terms altogether to describe mental health problems and may not recognise that being asked whether they have ‘felt down’ or have ‘lost interest in doing things’ is an enquiry into their mental health, particularly if they are not conversant with the language. In addition, women from ethnic minority backgrounds are less likely to report mental health problems due to a multitude of reasons, including perceived stigma and cultural norms [17, 18, 31], and it is possible that failure to report problems may be reflected in their recollection of being asked about those problems at all.

Aside from ethnicity, the current findings provide some evidence to suggest that women living in less socioeconomically advantaged areas and without or separately from a partner were less likely to be asked about their mental health during the perinatal period. However, the evidence is inconclusive and the association between these characteristics and women’s access to mental health support requires further exploration.

### Clinical implications

The women who are most at risk of perinatal mental health problems, such as women from ethnic minority backgrounds and women living in less socioeconomically advantaged areas [32], are the women who are least likely to be asked about their mental health. If the women most at risk are less likely to be asked about their mental health, they are less likely to report mental health concerns, less likely to be referred to specialist mental health services and less likely to receive treatment in order to recover. This places these women, their babies and their families at risk of adverse outcomes in the short and longer term and is consistent with the inverse care law, that “availability of good medical care tends to vary inversely with the need for it in the population served” [33]. This is also likely to further widen existing health disparities.

The first step to tackling this problem is to provide all women, regardless of background and circumstances, with the opportunity and encouragement to report any mental health concerns. Routinely asking about mental health not only provides the opportunity for disclosure, but also normalises and validates any problems experienced during the perinatal period. It is crucial that any barriers to asking women about their mental health are identified and addressed. For example, lack of expertise, cultural awareness and confidence amongst healthcare professionals can be overcome with adequate information and training; concerns about intrusive questioning can be reduced with the standardisation and frequency of such conversations and with improved rapport and trust through continuity of care; and language barriers can be tackled with increased availability of and use of professional interpreters, the allocation of adequate time to discuss mental as well as physical wellbeing, and greater cultural responsiveness in overall care so that healthcare professionals better reflect the communities they work in. If women are clearly and sensitively asked about their mental health and general wellbeing at every antenatal and postnatal appointment, as specified in the NICE guideline, fewer women will go unchecked and fewer mental health problems will be missed. Unless we overcome the barriers and improve performance in antenatal and postnatal mental health screening, disparities in access to mental health support and perinatal mental health outcomes are likely to widen.

### Strengths and limitations

The strengths of this study include the large samples of women recruited for each survey and the application of survey weights to increase the representativeness of the survey findings. In addition, the availability of comparative data from three consecutive time points allows us to monitor changes over time and, in particular, to evaluate the impact of the NICE guidelines published in 2014 and the 2020 Covid-19 pandemic on clinical practice. The main limitation of the study is the reliance on self-reported data, which is dependent on women’s recollection of events occurring some months previously. The 2014 survey recruited women who were three months postpartum and the 2018 and 2020 surveys recruited women who were six months postpartum. Therefore, women may have forgotten whether or not they were asked about their mental health at their antenatal booking and postnatal check appointments. Furthermore, women with mental health problems may be even less likely to recall or report being asked. In addition, due to variations in focus and in the specific questions included in different surveys, the analysis was limited to whether women were asked about their mental health and does not provide insight into women’s offer of or engagement with mental health support services.

## Conclusions

The results of this analysis are highly relevant to existing policy and future policy advocacy and planning. The NICE guideline is clear that healthcare professionals should discuss mental health and wellbeing with all women during pregnancy and the postnatal period. However, this analysis demonstrates both inadequate coverage of mental health screening questions during and after pregnancy, and sociodemographic disparities in this screening and therefore in symptoms being identified and access to onward services and support. The disparities observed in this study suggest that those women most likely to need support and treatment are the least likely to be supported in accessing it and may therefore be at greater risk of adverse outcomes. This could be improved by better training for GPs, midwives and other maternity healthcare professionals, more culturally responsive care, increased standardisation and frequency of screening questions, clear care pathways and better continuity of care.

## Supplementary Information

**Additional file 1.**

## Data Availability

The datasets used and/or analysed during the current study are available from the corresponding author on reasonable request.
