# Supplementary material for: Disparities in who is asked about their perinatal mental health: an analysis of cross-sectional data from consecutive national maternity surveys
Source: BMC Pregnancy Childbirth. 2023 Apr 27;23:263. doi: 10.1186/s12884-023-05518-4 (PMC10132923; doi:10.1186/s12884-023-05518-4)
Supplement: Supplementary file 1 — Additional file 1. [file 12884_2023_5518_MOESM1_ESM.docx]

**Supplementary File 1**

**Brief mental health screening questions and response options across the surveys**

|  | 2014 survey | 2018 survey | 2020 survey |
| --- | --- | --- | --- |
| Antenatal | At the time of your pregnancy booking or a few weeks after, were you asked about your emotional and mental health?  Response options: Yes, No | Around the time of your pregnancy booking, were you asked about your emotional and mental health?  Response options: Yes, No, Don’t Know | Around the time of your pregnancy booking, were you asked about your emotional and mental health?  Response options: Yes, No, Don’t Know |
|  |  |  |  |
|  | At the time of your pregnancy booking or a few weeks after, were you asked about your past mental health / family history?  Response options: Yes, No | Around the time of your pregnancy booking, were you asked about your past mental health or family history of mental health problems?  Response options: Yes, No, Don’t Know | Around the time of your pregnancy booking, were you asked about your past mental health or family history of mental health problems?  Response options: Yes, No, Don’t Know |
| Postnatal | Since your baby was born have you been asked about your emotional and mental health?  Response options: Yes, No | Since your baby was born have you been asked about your emotional and mental health by a health professional?  Response options: Yes, No, Don’t Know | Since your baby was born have you been asked about your emotional and mental health by a health professional?  Response options: Yes, No, Don’t Know |
